# Real-world management of hypercholesterolemia in patients after acute coronary syndrome in Greece^☆^

**DOI:** 10.1016/j.athplu.2025.03.002

**Published:** 2025-03-24

**Authors:** Despoina Massia, Periklis Giovas, Nikolaos Papadopoulos, Georgios Katsimagklis, Evangelos Pissimisis, Sotirios Patsilinakos, Evgenia Pappa, Giannis Baltogiannis, Nikolaos Kouremenos, Christos Dontas, Evangelos Liberopoulos

**Affiliations:** a Amgen Hellas, Athens, Greece; b Private Practice, Thessaloniki, Greece; c Department of Cardiology, Athens Naval Hospital, Athens, Greece; d Department of Cardiology, Tzanio Hospital, Piraeus, Greece; e Cardiology Department, General Hospital of Nea Ionia "Konstantopoulio," Nea Ionia, Greece; f Department of Cardiology, General Hospital "G. Hatzikosta," Ioannina, Greece; g Agios Loukas Hospital, Thessaloniki, Greece; h VUB Brussels, Brussels, Belgium; i Glyfada Med Private Clinic, Athens, Greece; j First Department of Propaedeutic Internal Medicine, Medical School, Laiko General Hospital, National and Kapodistrian University of Athens, Athens, Greece

**Keywords:** Acute coronary syndrome, Posthospitalization management, Real-world, Hypercholesterolemia

## Abstract

**Background:**

Prompt initiation of lipid-lowering therapy (LLT) following acute coronary syndrome (ACS) is crucial for preventing secondary cardiovascular events. However, there are gaps in clinical implementation of the 2019 ESC/EAS guideline-recommended low-density lipoprotein cholesterol (LDL-C) goal of <55 mg/dL in patients post-ACS.

**Methods:**

This multicenter, real-world, retrospective, 12-month study of adult patients in Greece hospitalized for ACS from September 2019 to November 2022 assessed the attainment of target LDL-C (<55 mg/dL) during the first year post-ACS. Eligible patients had elevated LDL-C at hospitalization (>130 mg/dL if LLT naïve; >100 mg/dL if on statin monotherapy; >70 mg/dL if on a statin plus ezetimibe) and ≥1 LDL-C measurement within 12 months post-ACS.

**Results:**

Overall, 212 eligible patients of mean (SD) age 59.9 (±11.1) years were enrolled. Type 2 diabetes and hypertension were reported in 19.8 % (42/212) and 50.9 % (108/212) of patients, respectively. Median (Q1, Q3) LDL-C was 138.0 (106.5, 158.0) mg/dL at hospitalization (n = 212). In patients with LDL-C availability at 12 months posthospitalization (n = 197), median (Q1, Q3) LDL-C was 64.0 (53.0, 76.0) mg/dL, with 27.9 % of patients (55/197) attaining LDL-C <55 mg/dL. Although 73.9 % of patients (199/212) were discharged from the hospital on statin monotherapy, 50 % of patients (106/212) were receiving statin-ezetimibe LLT and 1.4 % (3/212) were receiving statin-ezetimibe-PCSK9 inhibitor LLT 12 months posthospitalization.

**Conclusion:**

LDL-C goal attainment is suboptimal in the first year after ACS hospitalization in Greece, indicating an unmet need to improve the treatment of patients with hypercholesterolemia during the post-ACS period by optimizing lipid management through earlier LLT intensification.

## Highlights

• LDL-C goal attainment is suboptimal 1 year after ACS hospitalization in Greece.
• Most patients were discharged on statin monotherapy after an ACS event.
• Median LDL-C decreased from 138 to 64 mg/dL over a year of follow-up.
• Only 27.9 % of patients achieved the recommended target LDL-C of <55 mg/dL.
• There is an unmet need for earlier intensification of lipid-lowering therapy.

## 1 Introduction

Cardiovascular disease (CVD) is the leading cause of mortality and disability in Europe [[1], [2], [3]]. Acute coronary syndrome (ACS) represents the leading cause of CVD-related mortality and is often the first clinical manifestation of CVD [4]. Poorly controlled low-density lipoprotein cholesterol (LDL-C) is one of the major drivers and modifiable risk factors for CVD and is associated with a high risk of recurrent cardiovascular (CV) events [5]. The 2019 European Society of Cardiology/European Atherosclerosis Society (ESC/EAS) dyslipidemia management guidelines recommend attaining an LDL-C target of <55 mg/dL and a 50 % LDL-C reduction from baseline for very-high-CV-risk patients [5]. Registries and real-world studies, including DA VINCI, DYSIS, DYSIS II, EUROASPIRE, and SANTORINI, have demonstrated a gap between the guidelines and clinical lipid management across Europe; this has not only persisted but widened with the newer, more stringent guidelines [[6], [7], [8], [9], [10], [11], [12], [13], [14]]. Therefore, LDL-C goal attainment is suboptimal, with LDL-C values above guideline-recommended levels in both primary and secondary CV prevention for the majority of high- and very-high-CV-risk patients across the European Union [[6], [7], [8], [9], [10], [11]]. Contributory factors may include CV risk underestimation, poor adherence to prescribed treatment, or underutilization of the available treatment armamentarium due in part to local reimbursement guidelines [[6], [7], [8]]. Particularly for ACS patients, early initiation of intensive lipid-lowering therapy (LLT) is crucial for reducing the risk of early recurrent ischemic events [15], which is higher in the first year post-ACS hospitalization [16]. The recommended therapeutic options include statins alone or in combination with ezetimibe and proprotein convertase subtilisin/kexin type 9 inhibitors (PCSK9is) [5,17].

Greece has historically been classified as a low-CVD-risk country, but recently there has been an alarming increase in prevalence of established and emerging CVD risk factors [18]. Over 25 % of Greek adults with ≥1 CV risk factor are at high CV risk [19]. The annual incidence of ACS in Greece was 22.6 per 10,000 persons in 2003–2004, with an in-hospital mortality rate of 4.3 %; the in-hospital mortality rate for patients with ACS in 2020 was estimated to be 4.7 % [20,21]. Various national observational studies conducted during the previous decades have provided epidemiological data, identified gaps in the management of ACS patients, and confirmed that LDL-C goal attainment in this high-risk patient group remains suboptimal despite lifestyle modifications and in-hospital ACS management improvements over the years [[21], [22], [23], [24], [25], [26], [27]].

The aim of this study was to provide information related to the overall therapeutic management of ACS patients in Greece in the first 12 months post-ACS, which is the most critical period for readmissions due to recurrent CV events. The effectiveness of prescribed LLT in achieving the therapeutic targets set by the ESC/EAS 2019 guidelines in a real-world setting was also investigated. To the best of our knowledge, this is the first retrospective study in Greece to evaluate the post-ACS lipid management of patients in both outpatient clinics and private practices in the first 12 months post-ACS.

## 2 Methods

### 2.1 Study population

This was a retrospective multicenter study in Greece. Eligible patients were recruited from 27 outpatient cardiology hospital clinics and private practices. The data were derived from patient medical records at participating study sites. The participating investigators consecutively included all eligible adult patients from their archives who were hospitalized for ACS between September 2019 (when the ESC/EAS 2019 guidelines came into effect) and November 2022 who had a total follow-up of 12 months after ACS discharge and a full lipid profile available within 5 days of hospital admission for ACS. Eligible patients had LDL-C levels of >130 mg/dL if LLT naïve, >100 mg/dL if on statin monotherapy, or >70 mg/dL if on a statin plus ezetimibe at the time of admission for ACS. Patients who participated in an interventional clinical trial between ACS discharge and the 12 months following hospital discharge were excluded from the study.

Informed consent was obtained from each patient. The study was carried out according to the ethical principles for medical research involving human subjects established by the 1975 Declaration of Helsinki, protecting the privacy and confidentiality of personal information of all participants as reflected in a priori approval by the institution's human research committee.

Demographic data and clinical characteristics, including relevant medical history, were collected from patient medical records at hospitalization. Baseline (i.e., the first visit following hospital discharge) and subsequent follow-up clinic visits occurred as per normal practice between ACS discharge and the end of the 12-month observation period and were included in the analyses regardless of full lipid profile availability. These visits could be either onsite or remote as long as they were well documented in the medical records.

Patients were divided into subgroups for analysis based on whether they achieved the ESC/EAS 2019 guideline–recommended LDL-C target of <55 mg/dL in the 12 months following hospitalization for ACS. Group A achieved LDL-C <55 mg/dL within 12 months of hospital discharge, while group B had LDL-C levels still ≥55 mg/dL in this same period. Cholesterol measurement techniques varied by site and were not reported but were likely reflective of standard clinical practice. Standard clinical practice is typically indirect calculation of LDL-C levels via the Friedewald formula [28].

### 2.2 Statistical analysis

Due to the descriptive nature of this study, no formal hypothesis testing was conducted. Statistical analyses were performed for the patient demographics, relevant medical history, and clinical characteristics captured at hospital admission. Continuous variables were presented in terms of mean (standard deviation [SD]), median (25th, 75th percentiles), and minimum and maximum values, while categorical variables were presented as absolute (n) and relative (%) frequencies. Relative frequencies were calculated based on valid nonmissing cases.

Medication adherence using prescription claims was determined for the 12 months following hospital admission via proportion of days covered (PDC), defined as the percent ratio of days that LLT was received divided by the total number of days for which the patient was eligible to receive the LLT, as per prescribing information. Based on PDC, patient distribution according to adherence level was determined using the following cutoffs: >80 % (high adherence), 60 %–80 % (intermediate adherence), or <60 % (low adherence). LLT regimen modifications were also captured throughout the 12 months following hospital discharge.

Subgroup analyses were performed depending on whether the recommended target of the LDL-C ESC/EAS 2019 guidelines was attained (group A: LDL-C <55 mg/dL) or not (group B: LDL-C ≥55 mg/dL) at 12 months post-ACS. LDL-C values were also collected at the time of hospitalization (within 5 days of admission) and the first clinical visit following discharge (baseline). Between-group comparisons were performed with Fisher's exact test (for categorical variables) or Student's t-test (for continuous variables). The level of statistical significance was set at 5 %.

A multivariate linear regression model was employed to explore predictors of LDL-C goal attainment. Univariate logistic regression was performed for all candidate variables, including demographic characteristics, medical history, lipid profile components, and LLTs. Only variables with a *P* value < 0.10 in the univariate analysis were considered for inclusion in the multivariate model. Clinically relevant variables, such as lipid levels at hospitalization and the type of ACS (ST-elevation myocardial infarction [STEMI], non–STEMI [NSTEMI], or unstable angina), were prioritized given their established association with lipid management and CV outcomes. Results are presented as odds ratios (ORs) with 95 % confidence intervals (CIs). Model fit was assessed using standard metrics, including goodness-of-fit tests, and potential multicollinearity was checked using variance inflation factors. A two-sided *P* value < 0.05 was considered statistically significant. Univariate regressions were also performed comparing demographic characteristics with individual lipid levels.

## 3 Results

A total of 217 patients were screened and 212 were eligible for participation. For 15 of the 212 patients, no LDL-C level was available at 12 months posthospitalization, so goal achievement could not be assessed. LDL-C values were available for 165 patients at the baseline visit (i.e., the first visit following hospital discharge) and for 197 patients at 12 months posthospitalization (Supplemental Fig. 1).

The patient demographics and anthropometric characteristics are listed in Table 1 for the total study population and per group. The relevant medical history and ACS diagnosis at hospitalization are listed in Supplemental Tables 1 and 2. Type 2 diabetes and hypertension were reported in 19.8 % (42/212) and 50.9 % (108/212) of patients, respectively. Of the 212 patients, 90 (42.5 %) were on statin monotherapy and 7 (3.3 %) were on combination statin-ezetimibe therapy prior to the index ACS event. The mean (SD) duration of hospitalization was 5.3 (±3.6) days (Supplemental Table 2). The first visit after hospital discharge (i.e., baseline visit) took place at a median (minimum, maximum) of 133.0 (5.0, 725.0) days (Supplemental Table 3). Overall, 27.9 % (55/197) of patients with available data at 12 months post-ACS attained LDL-C <55 mg/dL (Table 2); these patients were considered to be in group A. There were no significant differences between groups A and B regarding demographics, past medical history, or hospitalization details (Supplemental Tables 1 and 2). Cardiac disorders (e.g., cardiac failure, arrhythmia, coronary artery disease) at index hospitalization were numerically more prevalent in group B, but group A had a numerically higher overall burden of comorbid medical conditions (Supplemental Table 1). A higher percentage of patients in group A than in group B had STEMI (47.3 % vs. 35.2 %). Accordingly, NSTEMI was more frequently observed among patients in group B than in group A (41.5 % vs. 21.8 %; Supplemental Table 2).

**Table 1:** Demographic, anthropometric, and lifestyle characteristics of patients at hospitalization.

	Total number of patients N = 212	Group A^a^: LDL-C <55 mg/dL n = 55	Group B^a^: LDL-C ≥55 mg/dL n = 142	*P* value for difference between groups^b^
**Age (years)**				
Mean (SD)	59.91 (11.11)	60.93 (11.64)	59.37 (11.10)	0.38
Median [Q1, Q3]	60.2 [51.1, 68.2]	61.0 [51.6, 70.9]	59.8 [51.1, 67.4]	
Minimum–maximum	39.2–89.1	40.6–80.5	39.2–89.1	
**Gender, n (%)**				
Male	173 (81.6)	47 (85.5)	112 (78.9)	0.32
Female	39 (18.4)	8 (14.5)	30 (21.1)	
**Ethnicity, n (%)**				
Caucasian	210 (99.1)	55 (100.0)	140 (98.6)	–
Black or African American	1 (0.5)	0 (0.0)	1 (0.7)	
Asian	1 (0.5)	0 (0.0)	1 (0.7)	
**BMI (kg/m^2^)**				
Mean (SD)	27.8 (4.2)	28.0 (4.1)	27.8 (4.4)	0.77
Median [Q1, Q3]	27.4 [25.2, 30.1]	27.4 [24.8, 30.7]	27.7 [25.3, 30.1]	
Minimum–maximum	17.6–43.2	21.6–40.1	17.6–43.2	
**Smoking status, n (%)**				
Nonsmoker	83 (39.2)	22 (40.0)	59 (41.6)	0.71
Ex-smoker	61 (28.8)	17 (30.9)	36 (25.4)	
Current smoker	68 (32.1)	16 (29.1)	47 (33.1)	

BMI, body mass index; LDL-C, low-density lipoprotein cholesterol; Q, quartile; SD, standard deviation.

a N values for groups A and B are reduced by a total of 15 (from 212 to 197) as 14 patients attending at least 1 visit post-ACS hospitalization did not provide any LDL-C measurements while 1 additional patient did not attend any visits.

b Between-group comparisons were performed with Fisher's exact test for categorical variables and Student's t-test for continuous variables.

**Table 2:** Attainment of LDL-C cutoffs during the first 12 months post-ACS hospitalization.

**Categorization of LDL-C**	**N = 212**
LDL-C <55 mg/dL	55 (27.9 %)
LDL-C ≥55 mg/dL	142 (72.1 %)
LDL-C <70 mg/dL	118 (59.9 %)
LDL-C 70–100 mg/dL	64 (32.5 %)
LDL-C >100 mg/dL	15 (7.6 %)
Missing^a^	15

ACS, acute coronary syndrome; LDL-C, low-density lipoprotein cholesterol.

a Fourteen patients attending at least 1 visit posthospitalization did not provide any LDL-C measurements. One additional patient did not attend any visits.

The mean (SD) LDL-C values for the whole cohort were 137.3 (±41.4) mg/dL at hospitalization (n = 212) and 71.7 (±26.3) mg/dL at the baseline visit (n = 165). In patients with available LDL-C at 12 months posthospitalization (n = 197), the mean LDL-C value was 67.6 (±23.3) mg/dL (Supplemental Table 4). For group A, the mean (SD) LDL-C values were 124.4 (±31.8) mg/dL at hospitalization (n = 55), 55.1 (±25.4) mg/dL at the baseline visit (n = 45), and 44.5 (±8.7) mg/dL at the 12-month time point (n = 55). For group B, the corresponding values were 142.7 (±44.5) mg/dL at hospitalization (n = 142), 77.9 (±23.9) mg/dL at the baseline visit (n = 120), and 76.6 (±20.9) mg/dL at the 12-month time point (n = 142). The mean (SD) distance from the ESC/EAS 2019–recommended LDL-C goal (<55 mg/dL) was −10.5 (±8.7) and +21.6 (±20.9) mg/dL for patients in groups A and B, respectively (Supplemental Table 5).

At hospital discharge, most patients (93.9 %) were receiving statin monotherapy, whereas approximately 50 % of patients had modified treatment to statin-ezetimibe combination therapy at 12 months post-ACS hospitalization. Combination LLT including a PCSK9i was prescribed in only 3 patients, 2 of whom had available LDL-C data at study end and attained the LDL-C <55 mg/dL target (Table 3). Patient adherence to LLT treatment was high (>80 %) for over 97 % of patients overall and was similar between groups A and B (Supplemental Table 6). LLT regimens were adjusted mainly via the addition of non-statin therapy to existing treatment (rather than switching statins or increasing statin intensity), with approximately 60 % of patients having one non-statin therapy added over the course of a year (Supplemental Table 7).

**Table 3:** Lipid-lowering treatment received after hospital discharge and during the 12-month post-ACS hospitalization follow-up.

	Total number of patients N = 212	Group A^a^: LDL-C <55 mg/dL n = 55	Group B^a^: LDL-C ≥55 mg/dL n = 142
**LLT after hospital discharge**			
Low-intensity statin monotherapy, n (%)	8 (3.8 %)	3 (5.5 %)	4 (2.8 %)
Moderate-intensity statin monotherapy, n (%)	33 (15.6 %)	7 (12.7 %)	23 (16.2 %)
High-intensity statin monotherapy, n (%)	158 (74.5 %)	42 (76.4 %)	106 (74.7 %)
Ezetimibe monotherapy, n (%)	10 (4.7 %)	2 (3.6 %)	7 (4.9 %)
PCSK9i monotherapy, n (%)	3 (1.4 %)	1 (1.8 %)	2 (1.4 %)
***P* value to assess differences between groups**^b^	–	0.82	
**At follow-up**			
Low-intensity statin monotherapy, n (%)	6 (2.8 %)	3 (5.5 %)	3 (2.1 %)
Moderate-intensity statin monotherapy, n (%)	19 (9.0 %)	3 (5.5 %)	15 (10.6 %)
High-intensity statin monotherapy, n (%)	78 (36.8 %)	17 (30.9 %)	55 (38.7 %)
Ezetimibe monotherapy, n (%)	1 (0.5 %)	1 (1.8 %)	0 (0.0 %)
Statin + ezetimibe combination, n (%)	103 (48.6 %)	28 (50.9 %)	68 (47.9 %)
PCSK9i combination,^c^ n (%)	3 (1.4 %)	2 (3.6 %)	0 (0.0 %)
Other, n (%)	2 (0.9 %)	1^d^ (1.8 %)	1^d^ (0.7 %)
***P* value to assess differences between groups**^b^	–	0.06	

ACS, acute coronary syndrome; LDL-C, low-density lipoprotein cholesterol; LLT, lipid-lowering therapy; PCSK9i, proprotein convertase subtilisin/kexin type 9 inhibitor.

a N values for groups A and B are reduced by a total of 15 (from 212 to 197) as 14 patients attending at least 1 visit posthospitalization did not provide any LDL-C measurements while 1 additional patient did not attend any visits.

b The groups were compared using Fisher's exact test.

c Combined with either statin + ezetimibe (n = 2) or ezetimibe alone (n = 1). One of the 3 patients under PCSK9i combination therapy did not have available LDL-C at 12 months post-ACS hospitalization and could not be classified under group A or B.

d Fenofibrate (n = 1).

Few patients (16.5 % of the study population; 21.8 % of group A and 16.2 % of group B) had CVD-related hospitalizations, emergency room visits, or outpatient visits within 12 months of their first ACS hospitalization (Supplemental Table 8). The most frequently reported reasons for subsequent hospitalizations were angiography for group A (35.7 % of admissions) and planned percutaneous coronary intervention for group B (39.1 % of admissions).

A linear regression analysis was performed to explore independent relationships between lipid profile components at the study end and other patient characteristics. Significant relationships were found between final LDL-C level and LDL-C, total cholesterol, and non–high-density lipoprotein cholesterol levels at hospitalization and the baseline visit, as well as baseline triglyceride levels (Supplemental Table 9). Logistic regression analysis (multivariate and univariate) was conducted to identify predictors of LDL-C target attainment (Table 4). LDL-C at hospitalization (multivariate OR 0.985; 95 % CI 0.975, 0.995; *P* = 0.003) and STEMI (multivariate OR 3.816; 95 % CI 1.635, 8.911; *P* = 0.002) emerged as significant predictors in both univariate and multivariate regression analyses.

**Table 4:** Logistic regression analysis to explore potential predictors of LDL-C <55 mg/dL target attainment.

Explanatory variable	Univariate analysis OR [(95 % CI), *P* value]	Multivariable analysis^a^ OR [(95 % CI), *P* value]
**Age in years (n=197)**	1.012^b^ [(0.985, 1.041), 0.38]	–
**BMI in kg/m^2^ (n=197)**	1.011^b^ [(0.940, 1.087), 0.77]	–
**Gender**		–
Female (n = 39)	0.636^c^ [(0.271, 1.489), 0.30]	
Male (n = 173)	1	
**Hypertension**		–
Yes (n = 107)	0.872^c^ [(0.467, 1.626), 0.67]	
No (n = 105)	1	
**Type 2 diabetes**		–
Yes (n = 42)	1.136^d^ [(0.530, 2.434), 0.74]	
No (n = 170)	1	
**Smoking status**		–
Current smoker (n = 68)	0.913^c^ [(0.432, 1.932), 0.81]	
Ex-smoker (n = 61)	1.266^d^ [(0.594, 2.699), 0.54]	
Nonsmoker (n = 83)	1	
**ACS type**		
STEMI (n = 80)	2.557^d^ [(1.171, 5.582), 0.02]	3.816^d^ [(1.635, 8.911), 0.002]
Unstable angina (n = 53)	2.458^c^ [(1.037, 5.829), 0.04]	2.271^c^ [(0.921, 5.599), 0.07]
Non-STEMI (n = 77)	1	1
**Lipid profile components measured at ≤5 days from hospital admission**		
LDL-C (n = 197)	0.988^e^ [(0.979, 0.997), 0.01]	0.985^e^ [(0.975, 0.995), 0.003]
HDL-C (n = 197)	0.976^e^ [(0.948, 1.006), 0.11]	–
Triglycerides (n = 197)	1.001^b^ [(0.997, 1.005), 0.52]	–
Total cholesterol (n = 197)	0.990^e^ [(0.983, 0.998), 0.01]	–
Non–HDL-C (n = 197)	0.992^e^ [(0.984, 0.999), 0.03]	–
**Lipid profile components measured at baseline**		
LDL-C (n = 165)	0.933^e^ [(0.908, 0.959), <0.001]	–
HDL-C (n = 165)	1.004^b^ [(0.974, 1.036), 0.78]	–
Triglycerides (n = 165)	0.997^e^ [(0.989, 1.004), 0.35]	–
Total cholesterol (n = 165)	0.963^e^ [(0.945, 0.981), <0.001]	–
Non–HDL-C (n = 165)	0.961^e^ [(0.942, 0.980), <0.001]	–
**Lipid profile components measured at study end**		
LDL-C (n = 197)	0.133^e^ [(0.031, 0.562), <0.001]	–
HDL-C (n = 197)	0.975^e^ [(0.944, 1.006), 0.11]	–
Triglycerides (n = 197)	1.001^b^ [(0.994, 1.006), 0.98]	–
Total cholesterol (n = 197)	0.913^e^ [(0.888, 0.939), <0.001]	–
Non–HDL-C (n = 197)	0.901^e^ [(0.872, 0.932), <0.001]	–
**LLT received during study**		
Statin + additional medication^f^ (n = 132)	1.130^d^ [(0.588, 2.172), 0.71]	1.127^d^ [(0.557, 2.280), 0.74]
Statin monotherapy (n = 79)	1	1

ACS, acute coronary syndrome; BMI, body mass index; CI, confidence interval; HDL-C, high-density lipoprotein cholesterol; LDL-C, low-density lipoprotein cholesterol; LLT, lipid-lowering therapy; OR, odds ratio; PCSK9i, proprotein convertase subtilisin/kexin type 9 inhibitor; STEMI, ST-elevation myocardial infarction.

a Model included LLT therapy, ACS type, and LDL-C measured at ≤5 days from hospital admission.

b Indicates [(OR – 1) × 100] % increase in the odds of achieving LDL-C <55 mg/dL for a unit increase in the explanatory variable.

c Indicates [(1 – OR) × 100] % lower odds of achieving LDL-C <55 mg/dL compared to the reference category (i.e., category = 1).

d Indicates [(OR – 1) × 100] % higher odds of achieving LDL-C <55 mg/dL compared to the reference category (i.e., category = 1).

e Indicates [(1 – OR) × 100] % reduction in the odds of achieving LDL-C <55 mg/dL for a unit increase in the explanatory variable.

f Ezetimibe and/or PCSK9i.

## 4 Discussion

This study provides insights into the lipid management of post-ACS patients with hypercholesterolemia in Greece in light of the stricter therapeutic LDL-C targets proposed by the 2019 ESC/EAS guidelines. Patient demographics, lifestyle characteristics, and prevalence of risk factors were comparable to those of other studies in hospitalized ACS patients, despite variations in eligibility criteria [[20], [21], [22], [23], [24], [25], [26]]. Most patients were male, overweight, and either nonsmokers or ex-smokers at hospitalization. The mean length of hospital stay was comparable to that in other national studies, as was the prevalence of STEMI and NSTEMI [21,23].

LDL-C target attainment (<55 mg/dL) was suboptimal; only 27.9 % of patients with data availability at 12 months post-ACS attained LDL-C <55 mg/dL. Contributing factors may include LLT not being initiated or intensified promptly as directed by clinical guidelines (clinical inertia) and poor adherence to treatment [29]. However, this study reported that adherence to LLT was high, which confirms data from other studies that have shown high treatment adherence but low rates of goal attainment [25,30,31].

In looking for predictors of LDL-C goal achievement, this study found no significant difference in the overall prescribed treatment patterns between groups A and B at hospital discharge or by study end, and no significant difference in treatment adherence. The first follow-up visit occurred sooner in group A than in group B, though not significantly (median time 99.0 vs. 133.5 days). In agreement with the literature, higher LDL-C levels at admission are a predictor of not attaining an LDL-C target at 12 months post-ACS hospitalization [32]. We also found that the index ACS event being STEMI, which may be associated with greater rates of cardiogenic shock than NSTEMI [33], was predictive of goal attainment; therefore, the interplay between the STEMI and NSTEMI pathophysiological mechanisms and LDL-C levels may merit further investigation.

Most study patients received high-intensity statin therapy at hospital discharge (76.4 % of group A and 74.7 % of group B), which aligns with published data on prescription patterns in ACS patients from national and international studies [6,8,22,24,25,34,35]. These prescription patterns are also aligned with guideline recommendations to prescribe high-intensity LLT (statins or combination therapy) during the first days after hospitalization for ACS [36,37]. By study end, intensification of the prescribed LLT was observed, with approximately 50 % of the overall study population receiving statin-ezetimibe combination LLT. Although the percentage of patients on statin monotherapy after hospital discharge was comparable to that of the 2013–2014 Greek nationwide DYSIS II ACS cohort, this study showed higher rates of combination therapy use at follow-up (48.6 % in the present study vs. <10 % in DYSIS II ACS) [25]. Notably, only 25 % of patients in the DYSIS II ACS cohort attained the 2016 ESC/EAS goal of LDL-C <70 mg/dL at approximately 120 days after admission [25], whereas in the current study, 59.9 % of patients would have met this less stringent goal at 12 months post-ACS hospitalization. The increased use of combination therapy during the present study and the high adherence to treatment potentially contributed to the limited extent of CV-related complications and subsequent hospitalization [38].

Combination regimens containing PCSK9i were rarely administered either after hospital discharge or at 12 months post-ACS hospitalization, consistent with other observational studies across Europe in secondary CV prevention [[6], [7], [8]]. The limited use of PCSK9i reported in the current study may be associated with barriers in local reimbursement criteria [8,39], as during the study, PCSK9is were reimbursed only in the following groups if already on maximally tolerated statin therapy and ezetimibe: 1) patients with familial hypercholesterolemia and LDL-C ≥130 mg/dL; 2) patients with established CV disease and/or diabetes with chronic kidney disease or target organ damage and LDL-C ≥100 mg/dL; and 3) statin-intolerant patients with LDL-C ≥130 mg/dL if high risk or ≥100 mg/dL if very high risk [40].

Based on the distance from LDL-C target attainment, patients in group B would likely have benefited from the timely administration of add-on non-statin therapies, such as ezetimibe or PCSK9i, which could reduce LDL-C levels by a further 20 %–25 % and 50 %–60 % by study end, respectively [41]. Previous studies have repeatedly shown that earlier intensification of LLT, particularly the addition of PCSK9i, leads to better LDL-C goal attainment and reduces the risk of recurrent CV events after hospitalization for ACS [8,38,[42], [43], [44], [45], [46], [47], [48], [49], [50]]. Observational registry studies on ACS patients have also demonstrated that being on 3 or fewer secondary prevention medications (suboptimal medical therapy) or less intensive LLT regimens after hospital discharge were independent predictors of long-term mortality at 4 years [51]. As ΕSC/EAS 2019 guidelines recommend aggressive, early, and combined LLT in patients at very high CV risk [4], the lack of prescription of aggressive combination LLT schemes after hospitalization and at first follow-up could have contributed to the low percentage of patients that attained the LDL-C target at 12 months post-ACS hospitalization.

Prompt follow-up posthospitalization is important for evaluating treatment effectiveness and, if applicable, intensifying it to optimize LDL-C target attainment in addition to promoting medication adherence [52]. In the present study, the first follow-up visit post-ACS hospitalization (baseline visit) occurred at a median of approximately 3–4 months, which is relatively late compared with the recommended follow-up times of 4–6 weeks for this very-high-CV-risk patient group [4].

### 4.1 Limitations

This study was observational and retrospective in nature. Direct comparisons with the results of other cited studies are not feasible; nevertheless, trends can be observed. Missing data were anticipated but did not prevent assessment of the planned endpoints. Over 90 % of the patients had at least 1 lipid-relevant value recorded in their medical records after hospital discharge, and due to well-defined inclusion criteria, analysis of primary and secondary endpoints was possible. The final lipid-related entry from the medical records, which was used to determine LDL-C target attainment, was not obtained at a standardized time point. The medical management of ACS patients during hospitalization was not recorded, and follow-up LLT administration data were not captured at consistent intervals. This study was also conducted during the COVID-19 pandemic, which may have significantly influenced the intervals between follow-up visits. Adherence was determined from medical claims using PDC and not derived from direct patient reporting. PDC is commonly used to determine real-world prescription refill behavior with the aim to identify areas of unmet need and reveal potential opportunities for interventions to promote adherence [53]. However, PDC cannot be used to confirm that medication was administered.

The study population represented only the subset of patients with ACS meeting minimum LDL-C level inclusion criteria, which may limit the generalizability of the study results. Nevertheless, the recruitment of patients from hospital outpatient and private clinics distributed across a wide geographical region, under routine clinical practice conditions, contributed valuable real-world evidence. The consecutive enrollment of patients from medical records who met eligibility criteria limited patient selection bias. Furthermore, patients who were hospitalized before September 2019 were not included in this study, as treating physicians would have been targeting a less stringent LDL-C goal prior to the publication of the new ESC/EAS guidelines at that time.

The sample size of patients treated with combination therapies in this study was relatively small compared with that of patients on statin monotherapy, which limited the statistical power to detect significant differences in LDL-C target achievement. For example, a greater increase in goal attainment between patients on ezetimibe combination therapy compared with statin monotherapy would be expected based on results from the RACING trial, during which 42 % of patients taking ezetimibe but only 25 % of patients on statin monotherapy met the <55 mg/dL LDL-C goal vs. 29 % and 24 %, respectively, in the present study [54]. A larger sample size and methodologies like stabilized inverse probability of treatment weighting could better reveal significant benefits of combination therapy, including improved clinical outcomes [55].

## 5 Conclusion

Overall, this study demonstrated that patients hospitalized for ACS were prescribed appropriate LLT at discharge and demonstrated high rates of treatment adherence. Approximately 50 % were prescribed ezetimibe as an add-on therapy within the first year, a marked increase compared with previous studies in a Greek ACS population. However, lengthy follow-up times and a lack of PCSK9i prescription (likely due to reimbursement challenges) may have contributed to less than a third of patients meeting the recommended LDL-C goal of <55 mg/dL in the first year after ACS hospitalization in Greece. There is an unmet need to improve the treatment of patients with hypercholesterolemia during the post-ACS period by optimizing lipid management through earlier LLT intensification, which would likely improve LDL-C target attainment. Revising local reimbursement criteria, in alignment with ESC/EAS 2019 recommendations, could significantly contribute to LDL-C target attainment, thus bridging the gap between guidelines and routine clinical practice.

## Data availability

All relevant data from this trial are included in the article and Supplementary Appendix. Qualified researchers may request data from Amgen clinical studies. Complete details are available at the following: https://www.amgen.com/science/clinical-trials/clinical-data-transparency-practices/clinical-trial-data-sharing-request.

## Acknowledgment of grant support

This study was designed and funded by Amgen Hellas.

## Declaration of competing interest

The authors declare the following financial interests/personal relationships, which may be considered as potential competing interests:

Periklis Giovas reports that financial support was provided by Amgen Inc. Writing assistance was provided by Red Nucleus and funded by Amgen Inc. Despoina Massia reports a relationship with Amgen Inc that includes equity or stocks. Periklis Giovas reports a relationship with Amgen Inc that includes equity or stocks. Evangelos Liberopoulos reports relationships with Amgen Inc., AstraZeneca, Bayer, Boehringer Ingelheim, Lilly, Merck Sharp & Dohme, Novartis, Novo Nordisk, Sanofi, and Servier that include consulting or advisory and funding grants. All other authors declare that they have no known competing financial interests or personal relationships that could have appeared to influence the work reported in this paper.
