# Supplementary material for: Real-world management of hypercholesterolemia in patients after acute coronary syndrome in Greece
Source: Atheroscler Plus. 2025 Mar 24;60:20–6. doi: 10.1016/j.athplu.2025.03.002 (PMC11999335; doi:10.1016/j.athplu.2025.03.002)
Supplement: Multimedia component 1 [file mmc1.docx]

**Supplemental Figure 1. Study participants**

***
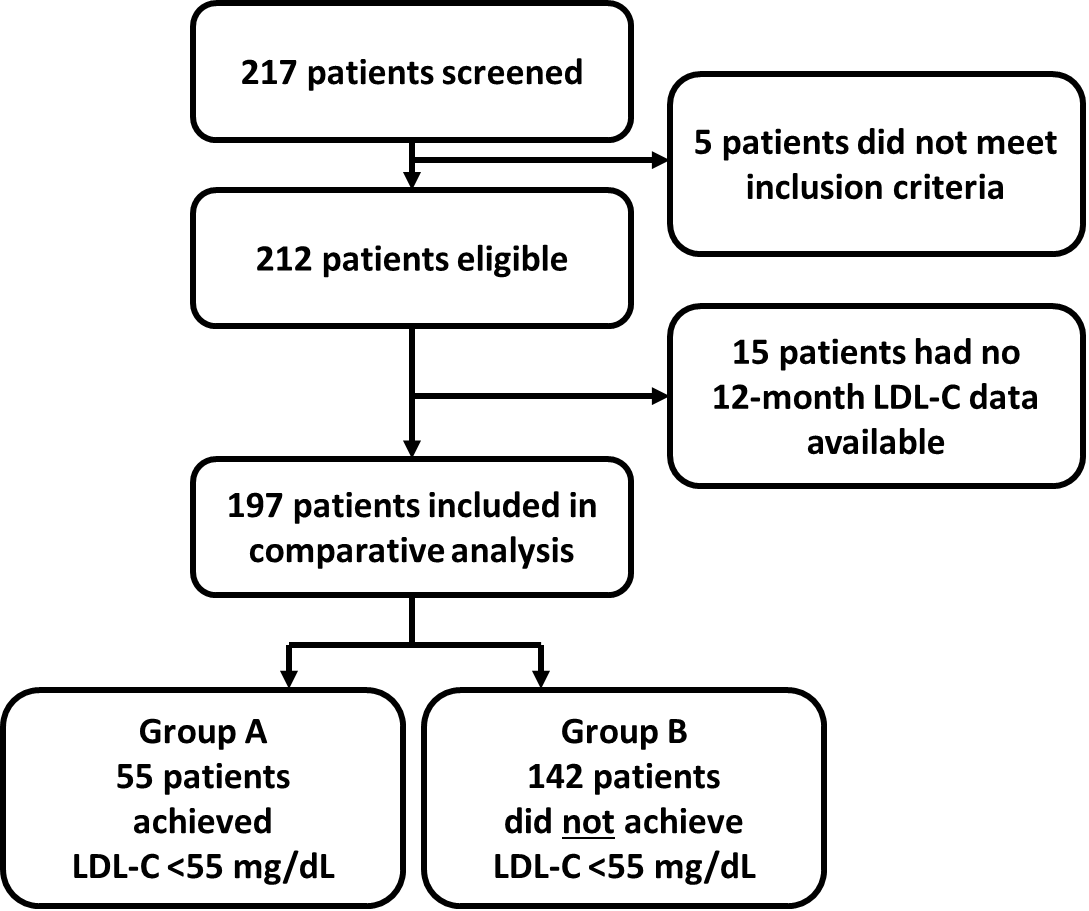
***

LDL-C, low-density lipoprotein cholesterol.

**Supplemental Table 1. Relevant medical history**

| **Relevant medical history** | **Total number of patients**  **N = 212** | **Group A^a^:**  **LDL-C <55 mg/dL**  **n = 55** | **Group B^a^:**  **LDL-C ≥55 mg/dL**  **n = 142** |
| --- | --- | --- | --- |
| **Does the patient have any relevant medical history?** | | | |
| Yes, n (%) | 170 (80.2) | 46 (83.6) | 111 (78.2) |
| No, n (%) | 42 (19.8) | 9 (16.4) | 31 (21.8) |
| *P* value to assess group differences | - | 0.44 | |
| **If yes, number of medical conditions, n (%)** | | | |
| 1 relevant medical history condition | 57 (33.5) | 11 (23.9) | 40 (36.0) |
| 2 relevant medical history conditions | 68 (40.0) | 24 (52.2) | 41 (36.9) |
| 3 relevant medical history conditions | 29 (17.1) | 8 (17.4) | 18 (16.2) |
| ≥4 relevant medical history conditions | 16 (9.4) | 3 (6.5) | 12 (10.8) |
| *P* value to assess group differences^b^ | - | 0.47 | |
| **CV-related conditions or risk factors reported in ≥5% of study patients,^c^** **n (%)** | | | |
| Hypertension | 108 (50.9) | 26 (47.3) | 72 (50.7) |
| Type 2 diabetes | 42 (19.8) | 12 (21.8) | 28 (19.7) |
| Arrhythmia | 35 (16.5) | 7 (12.7) | 25 (17.6) |
| Coronary artery disease | 22 (10.4) | 1 (1.8) | 18 (12.7) |
| Cardiac failure | 12 (5.7) | 2 (3.6) | 9 (6.3) |

ACS, acute coronary syndrome; CV, cardiovascular; LDL-C, low-density lipoprotein cholesterol.

^a^N values for groups A and B are reduced by a total of 15 (from 212 to 197) as 14 patients attending at least 1 visit post–ACS hospitalization did not provide any LDL-C measurements while 1 additional patient did not attend any visits after hospital discharge.

^b^The group means were compared using Fisher’s exact test.

^c^Verbatim terms reported by investigators; multiple conditions may have been reported per patient.

**Supplemental Table 2. Details of ACS hospitalization**

|  | **Total number of patients**  **N = 212** | **Group A^a^:**  **LDL-C <55 mg/dL**  **n = 55** | **Group B^a^:**  **LDL-C ≥55 mg/dL**  **n = 142** | ***P* value for difference between groups** |
| --- | --- | --- | --- | --- |
| **Type of ACS, n (%)** | | | | |
| STEMI | 80 (37.7) | 26 (47.3) | 50 (35.2) | 0.04^b^ |
| NSTEMI | 77 (36.3) | 12 (21.8) | 59 (41.5) |  |
| Unstable angina | 53 (25.0) | 16 (29.1) | 32 (22.5) |  |
| Unknown | 2 (0.9) | 1 (1.8) | 1 (0.7) |  |
| **Time from admission to discharge (in days)** | | | | |
| Mean (SD) | 5.27 (3.59) | 5.60 (3.84) | 5.35 (3.58) | 0.67^c^ |
| Median (Q1, Q3) | 5.0 (3.0, 7.0) | 6.0 (3.0, 8.0) | 5.0 (3.0, 6.0) |  |
| Minimum–maximum | 0.0–21.0 | 1.0–21.0 | 0.0–19.0 |  |

ACS, acute coronary syndrome; LDL-C, low-density lipoprotein cholesterol; NSTEMI, non–ST-elevation myocardial infarction; Q, quartile; SD, standard deviation; STEMI: ST-elevation myocardial infarction.

^a^N values for groups A and B are reduced by a total of 15 (from 212 to 197) as 14 patients attending at least 1 visit post–ACS hospitalization did not provide any LDL-C measurements while 1 additional patient did not attend any visits after hospital discharge.

^b^The group means were compared using Fisher’s exact test.

^c^The group means were compared using Student’s t-test.

**Supplemental Table 3. Time (in days) from ACS hospital discharge to first follow-up (baseline) visit**

|  | Total number of patients  N = 212 | Group A^a^:  LDL-C <55 mg/dL  n = 55 | Group B^a^:  LDL-C ≥55 mg/dL  n = 142 |
| --- | --- | --- | --- |
| **Time from hospital discharge to baseline visit (in days)** | | | |
| Mean (SD) | 165.95 (124.06) | 138.84 (121.29) | 162.79 (120.61) |
| Median (Q1, Q3) | 133.0 (56.0, 259.0) | 99.0 (38.0, 210.0) | 133.5 (59.0, 237.0) |
| Minimum–maximum^b^ | 5.0–725.0 | 15.0–520.0 | 5.0–725.0 |
| Missing | 1 | - | - |
| **Difference in group means**  **[(95% CI), *P* value]^c^** | - | –23.95 [(–61.79, 13.89), 0.21] | |
| ACS, acute coronary syndrome; CI, confidence interval; LDL-C, low-density lipoprotein cholesterol; Q, quartile; SD, standard deviation.  ^a^N values for groups A and B are reduced by a total of 15 (from 212 to 197) as 14 patients attending at least 1 visit post–ACS hospitalization did not provide any LDL-C measurements while 1 additional patient did not attend any visits after hospital discharge.  ^b^The follow-up for 5 patients exceeded 12 months.  ^c^The group means were compared using Student’s t-test. | | | |

**Supplemental Table 4. Lipid profile details at different study time points**

|  | **Total number of patients** | **Group A^a^:**  **LDL-C <55 mg/dL** | **Group B^a^:**  **LDL-C ≥55 mg/dL** | **Mean difference between groups**  **[(95% CI), *P* value]^b^** |
| --- | --- | --- | --- | --- |
| ***Lipid profile components measured at*** ***≤5 days from hospital admission (N = 212)*** | | | | |
| **LDL-C (mg/dL)** | | | | |
| Mean (SD, n) | 137.3 (41.4, 212) | 124.4 (31.8, 55) | 142.7 (44.5, 142) | 18.2 [(7.0, 29.5), 0.002] |
| Median (Q1, Q3) | 138.0 (106.5, 158.0) | 124.0 (97.0, 143.0) | 139.5 (109.0, 161.0) |  |
| Minimum–maximum | 71.0–342.0 | 72.0–227.0 | 71.0–342.0 |  |
| **HDL-C (mg/dL)** | | | | |
| Mean (SD, n) | 44.6 (11.5, 212) | 42.3 (9.6, 55) | 45.12 (11.9, 142) | 2.9 [(–0.4, 6.1), 0.1] |
| Median (Q1, Q3) | 43.0 (37.0, 50.0) | 40.0 (35.0, 45.0) | 44.0 (38.0, 50.0) |  |
| Minimum–maximum | 20.0–87.0 | 24.0–69.0 | 20.0–87.0 |  |
| **Triglycerides (mg/dL)** | | | | |
| Mean (SD, n) | 151.4 (78.3, 212) | 157.2 (74.7, 55) | 149.2 (78.7, 142) | –8.0 [(–31.9, 15.9), 0.51] |
| Median (Q1, Q3) | 132.5 (103.0, 177.5) | 138.0 (110.0, 194.0) | 130.0 (100.0, 177.0) |  |
| Minimum–maximum | 37.0–479.0 | 58.0–460.0 | 37.0–479.0 |  |
| **Total cholesterol (mg/dL)** | | | | |
| Mean (SD, n) | 209.5 (47.5, 212) | 195.4 (36.5, 55) | 214.4 (50.8, 142) | 19.0 [(6.2, 31.9), 0.004] |
| Median (Q1, Q3) | 204.0 (174.0, 237.5) | 197.0 (167.0, 218.0) | 210.5 (175.0, 244.0) |  |
| Minimum–maximum | 127.0–445.0 | 127.0–309.0 | 129.0–445.0 |  |
| **Non–HDL-C (mg/dL)** | | | | |
| Mean (SD, n) | 164.90 (45.8, 212) | 153.1 (34.9, 55) | 169.3 (49.2, 142) | 16.2 [(3.8, 28.5), 0.01] |
| Median (Q1, Q3) | 161.0 (131.5, 189.5) | 158.0 (130.0, 172.0) | 169.0 (134.0, 194.0) |  |
| Minimum–maximum | 82.0–378.0 | 84.0–266.0 | 82.0–378.0 |  |
| ***Lipid profile components measured at first follow-up visit post–ACS hospitalization (baseline visit, N = 165)*** | | | | |
| **LDL-C (mg/dL)** | | | | |
| Mean (SD, n) | 71.7 (26.3, 165) | 55.1 (25.4, 45) | 77.9 (23.9, 120) | 22.8 [(14.0, 31.5), <0.001] |
| Median (Q1, Q3) | 68.0 (57.0, 80.0) | 50.0 (42.0, 68.0) | 71.5 (62.5, 86.5) |  |
| Minimum–maximum | 25.0–201.0 | 25.0–191.0 | 36.0–201.0 |  |
| **HDL-C (mg/dL)** | | | | |
| Mean (SD, n) | 43.9 (11.1, 165) | 44.3 (12.7, 45) | 43.8 (10.5, 120) | –0.5 [(–4.4, 3.3), 0.80] |
| Median (Q1, Q3) | 42.0 (36.0, 51.0) | 42.0 (36.0, 49.0) | 43.0 (36.0, 51.5) |  |
| Minimum–Maximum | 22.0–88.0 | 27.0–88.0 | 22.0–74.0 |  |
| **Triglycerides (mg/dL)** | | | | |
| Mean (SD, n) | 122.5 (51.8, 165) | 116.4 (36.1, 45) | 124.8 (56.5, 120) | 8.5 [(–6.3, 23.2), 0.26] |
| Median (Q1, Q3) | 115.0 (83.0, 147.0) | 120.0 (82.0, 136.0) | 114.0 (83.5, 151.0) |  |
| Minimum–maximum | 34.0–388.0 | 59.0–195.0 | 34.0–388.0 |  |
| **Total cholesterol (mg/dL)** | | | | |
| Mean (SD, n) | 140.6 (29.0, 165) | 125.7 (27.9, 45) | 146.1 (27.5, 120) | 20.4 [(10.7, 30.1), <0.001] |
| Median (Q1, Q3) | 136.0 (123.0, 153.0) | 124.0 (109.0, 139.0) | 142.5 (126.5, 155.5) |  |
| Minimum–maximum | 76.0–285.0 | 76.0–256.0 | 94.0–285.0 |  |
| **Non–HDL-C (mg/dL)** | | | | |
| Mean (SD, n) | 96.7 (29.2, 165) | 81.4 (27.5, 45) | 102.4 (27.9, 120) | 20.9 [(11.3, 30.6), <0.001] |
| Median (Q1, Q3) | 91.0 (77.0, 109.0) | 75.0 (65.0, 91.0) | 94.5 (83.5, 115.5) |  |
| Minimum–maximum | 44.0–238.0 | 44.0–216.0 | 62.0–238.0 |  |
| ***Lipid profile components measured at 12 months post–ACS hospitalization (N = 197)*** | | | | |
| **LDL-C (mg/dL)** | | | | |
| Mean (SD, n) | 67.6 (23.3, 197) | 44.5 (8.7, 55) | 76.6 (20.9, 142) | 32.1 [(27.9, 36.2), <0.001] |
| Median (Q1, Q3) | 64.0 (53.0, 76.0) | 47.0 (40.0, 51.0) | 72.0 (62.0, 85.0) |  |
| Minimum–maximum | 18.0–186.0 | 18.0–54.0 | 55.0–186.0 |  |
| **HDL-C (mg/dL)** | | | | |
| Mean (SD, n) | 44.3 (10.2, 197) | 42.4 (10.9, 55) | 45.0 (9.9, 142) | 2.6 [(–0.8, 5.9), 0.13] |
| Median (Q1, Q3) | 43.0 (37.0, 51.0) | 40.0 (34.0, 48.0) | 44.0 (38.0, 52.0) |  |
| Minimum–Maximum | 23.0–78.0 | 27.0–78.0 | 23.0–70.0 |  |
| **Triglycerides (mg/dL)** | | | | |
| Mean (SD, n) | 119.9 (52.5, 197) | 119.8 (51.2, 55) | 119.9 (53.2, 142) | 0.17 [(–16.13, 16.47), 0.98] |
| Median (Q1, Q3) | 110.0 (85.0, 140.0) | 103.0 (89.0, 155.0) | 117.0 (82.0, 140.0) |  |
| Minimum–maximum | 34.0–399.0 | 34.0–313.0 | 41.0–399.0 |  |
| **Total cholesterol (mg/dL)** | | | | |
| Mean (SD, n) | 136.2 (27.5, 197) | 113.2 (17.3, 55) | 145.2 (25.4, 142) | 32.0 [(25.8, 38.3), <0.001] |
| Median (Q1, Q3) | 134.0 (120.0, 149.0) | 111.0 (100.0, 123.0) | 142.0 (129.0, 156.0) |  |
| Minimum–maximum | 83.0–267.0 | 83.0–159.0 | 105.0–267.0 |  |
| **Non–HDL-C (mg/dL)** | | | | |
| Mean (SD, n) | 92.0 (26.4, 197) | 70.8 (16.1, 55) | 100.2 (25.0, 142) | 29.5 [(23.5, 35.4), <0.001] |
| Median (Q1, Q3) | 88.0 (74.0, 105.0) | 70.0 (62.0, 77.0) | 94.0 (84.0, 111.0) |  |
| Minimum–maximum | 29.0–213.0 | 29.0–118.0 | 59.0–213.0 |  |

ACS, acute coronary syndrome; CI, confidence interval; HDL-C, high-density lipoprotein cholesterol; LDL-C, low-density lipoprotein cholesterol; Q, quartile; SD, standard deviation.

^a^N values for groups A and B are reduced by a total of 15 (from 212 to 197) as 14 patients attending at least 1 visit post–ACS hospitalization did not provide any LDL-C measurements while 1 additional patient did not attend any visits after hospital discharge.

^b^Mean difference is expressed as (group B – group A). The group means were compared using Student’s t-test.

**Supplemental** **Table 5. Level of achievement of the ESC/ΕΑS 2019 guideline–recommended LDL-C <55 mg/dL**

| **Difference between ESC/EAS 2019 LDL-C target and last observed LDL-C measurement (mg/dL)** | **Total number of patients**  **N = 212** | **Group A^a^:**  **LDL-C <55 mg/dL**  **n = 55** | **Group B^a^:**  **LDL-C ≥55 mg/dL**  **n = 142** |
| --- | --- | --- | --- |
| Mean (SD) | 12.6 (23.3) | –10.5 (8.7) | 21.6 (20.9) |
| Median (Q1, Q3) | 9.0 (–2.0, 21.0) | –8.0 (–15.0, –4.0) | 17.0 (7.0, 30.0) |
| Minimum, maximum | –37.0, 131.0 | –37.0, –1.0 | 0.0, 131.0 |

ACS, acute coronary syndrome; ESC/EAS, European Society of Cardiology/European Atherosclerosis Society; LDL-C, low-density lipoprotein cholesterol; Q, quartile; SD, standard deviation.

^a^N values for groups A and B are reduced by a total of 15 (from 212 to 197) as 14 patients attending at least 1 visit post–ACS hospitalization did not provide any LDL-C measurements while 1 additional patient did not attend any visits after hospital discharge.

**Supplemental** **Table** **6: Level of adherence to lipid-lowering therapy during the 12 months post–ACS hospitalization**

| **Level of adherence, n (%)** | **Total number of patients**  **N = 212** | **Group A^a^:**  **LDL-C <55 mg/dL**  **n = 55** | **Group B^a^:**  **LDL-C ≥55 mg/dL**  **n = 142** |
| --- | --- | --- | --- |
|  | | | |
| <60% | 4 (1.9) | 1 (1.8) | 3 (2.1) |
| 60%–80% | 1 (0.5) | 0 (0.0) | 1 (0.7) |
| >80% | 207 (97.6) | 54 (98.2) | 138 (97.2) |

ACS, acute coronary syndrome; LDL-C, low-density lipoprotein cholesterol.

^a^N values for groups A and B are reduced by a total of 15 (from 212 to 197) as 14 patients attending at least 1 visit post–ACS hospitalization did not provide any LDL-C measurements while 1 additional patient did not attend any visit after hospital discharge.

**Supplemental Table 7. Number of statin switches, add-ons, and titrations from ACS hospitalization up to 12 months post–ACS hospitalization**

|  | Total number of patients  N = 212 | Group A^a^:  LDL-C <55 mg/dL  n = 55 | Group B^a^:  LDL-C ≥55 mg/dL  n = 142 |
| --- | --- | --- | --- |
| **Number of statin switches** | | | |
| 0 | 170 (80.2%) | 45 (81.8%) | 112 (78.9%) |
| 1 | 35 (16.5%) | 10 (18.2%) | 23 (16.2%) |
| 2 | 7 (3.3%) | 0 (0.0%) | 7 (4.9%) |
| ***P* value to assess differences between groups**^b^ | - | 0.30 | |
| **Number of add-ons**^c^ | | | |
| 0 | 79 (37.3%) | 19 (34.6%) | 54 (38.0%) |
| 1^d^ | 132 (62.3%) | 36 (65.4%) | 88 (62.0%) |
| 2^e^ | 1^f^ (0.5%) | 0 (0.0%) | 0 (0.0%) |
| ***P* value to assess differences between groups**^b^ | - | 0.74 | |
| **Number of titrations**^g^ | | | |
| 0 | 170 (80.2%) | 41 (74.5%) | 116 (81.7%) |
| 1 | 40 (18.9%) | 14 (25.5%) | 24 (16.9%) |
| 2 | 2 (0.9%) | 0 (0.0%) | 2 (1.4%) |
| ***P* value to assess differences between groups**^b^ | - | 0.29 | |
| ACS, acute coronary syndrome; LDL-C, low-density lipoprotein cholesterol; PCSK9i, proprotein convertase subtilisin/kexin type 9 inhibitor.  ^a^N values for groups A and B are reduced by a total of 15 (from 212 to 197) as 14 patients attending at least 1 visit post–ACS hospitalization did not provide any LDL-C measurements while 1 additional patient did not attend any visits after hospital discharge.  ^b^The groups were compared using Fisher’s exact test.  ^c^Add-on is defined as a new medication given in addition to statin therapy.  ^d^Ezetimibe is given in addition to statin therapy.  ^e^Both ezetimibe and a PCSK9i are given in addition to statin therapy.  ^f^Patient did not provide any LDL-C measurements to be classified under group A or B.  ^g^Titration refers to a dose increase or decrease within the same statin. | | | |

**Supplemental** **Table 8. Details on CVD-related outpatient visits, emergency room visits, and hospitalizations during the first year after hospital discharge**

|  | **Total number of patients**  **N = 212** | **Group A^a^:**  **LDL-C <55 mg/dL**  **n = 55** | **Group B^a^:**  **LDL-C ≥55 mg/dL**  **n = 142** | ***P* value for difference between groups**^b^ |
| --- | --- | --- | --- | --- |
| Patients with CVD-related outpatient visits, emergency room visits, or hospitalizations over the first year after hospital discharge, n (%) | 35 (16.5) | 12 (21.8) | 23 (16.2) | 0.41 |
| ***Outpatient visits*** | | | | |
| Number of visits reported per patient, n (%) | | | | |
| 0 | 29 (82.9) | 11 (91.7) | 18 (78.3) | 0.81 |
| 1 | 4 (11.4) | 1 (8.3) | 3 (13.0) |  |
| 2 | 2 (5.7) | 0 (0.0) | 2 (8.7) |  |
| Reported reasons for outpatient visit, n (%) | | | | |
| Aortic aneurysm | 2 (25.0) | 0 (0.0) | 2 (28.6) | - |
| Ultrasound | 2 (25.0) | 0 (0.0) | 2 (28.6) |  |
| Holter | 2 (25.0) | 1 (100.0) | 1 (14.3) |  |
| Planned PCI | 1 (12.5) | 0 (0.0) | 1 (14.3) |  |
| Echocardiography & cardiac stress test | 1 (12.5) | 0 (0.0) | 1 (14.3) |  |
| ***Emergency room visits*** | | | | |
| Number reported per patient, n (%) | | | | |
| 0 | 32 (91.4) | 11 (91.7) | 21 (91.3) | 0.99 |
| 1 | 3 (8.6) | 1 (8.3) | 2 (8.7) |  |
| Reported reasons for emergency room visit, n (%) | | | | |
| Atrial fibrillation | 1 (33.3) | 0 (0.0) | 1 (50.0) | - |
| ACS | 1 (33.3) | 0 (0.0) | 1 (50.0) |  |
| Defibrillator implantation | 1 (33.3) | 1 (100.0) | - |  |
| ***Hospitalizations*** | | | | |
| Number reported per patient, n (%) | | | | |
| 0 | 5 (14.3) | 1 (8.3) | 4 (17.4) | 0.92 |
| 1 | 25 (71.4) | 9 (75.0) | 16 (69.6) |  |
| 2 | 3 (8.6) | 1 (8.3) | 2 (8.7) |  |
| 3 | 2 (5.7) | 1 (8.3) | 1 (4.3) |  |
| Reported reasons for hospitalization, n (%) | | | | |
| Planned PCI | 12 (32.4) | 3 (21.4) | 9 (39.1) | - |
| Angiography | 8 (21.6) | 5 (35.7) | 3 (13.0) |  |
| Coronary artery bypass grafting | 5 (13.5) | 2 (14.3) | 3 (13.0) |  |
| ACS | 4 (10.8) | 1 (7.1) | 3 (13.0) |  |
| Atrial fibrillation | 2 (5.4) | 0 (0.0) | 2 (6.5) |  |
| Aortic stenosis | 2 (5.4) | 0 (0.0) | 2 (6.5) |  |
| Defibrillator implantation | 1 (2.7) | 1 (7.1) | 0 (0.0) |  |
| Syncope | 1 (2.7) | 1 (7.1) | 0 (0.0) |  |
| ICD placement | 1 (2.7) | 1 (7.1) | 0 (0.0) |  |
| Valvular disease | 1 (2.7) | 0 (0.0) | 1 (3.3) |  |

ACS, acute coronary syndrome; CVD, cardiovascular disease; ICD, implantable cardioverter defibrillator; LDL-C, low-density lipoprotein cholesterol; PCI, percutaneous coronary intervention.

^a^N values for groups A and B are reduced by a total of 15 (from 212 to 197) as 14 patients attending at least 1 visit post–ACS hospitalization did not provide any LDL-C measurements while 1 additional patient did not attend any visit after hospital discharge.

^b^The groups were compared using Fisher’s exact test.

**Supplemental Table 9. Linear regression analysis to explore the independent relationship between lipid profile components at study end and other patient characteristics**

|  | **LDL-C**  **[(95% CI),**  ***P* value]** | **HDL-C**  **[(95% CI),**  ***P* value]** | **Triglycerides**  **[(95% CI),**  ***P* value]** | **Total cholesterol**  **[(95% CI),**  ***P* value]** | **Non–HDL-C**  **[(95% CI),**  ***P* value]** |
| --- | --- | --- | --- | --- | --- |
| **Age in years** | –0.24^a^ [(–0.53, 0.05), 0.10] | 0.19^a^  [(0.06, 0.31), 0.004] | –0.91^a^  [(–1.55, –0.26), 0.006] | –0.28^a^  [(–0.63, 0.06), 0.10] | –0.47^a^  [(–0.80, –0.15), 0.005] |
| **BMI in kg/m^2^** | 0.05^a^  [(–0.72, 0.82), 0.91] | –0.17^a^  [(–0.51, 0.16), 0.31] | 1.90^a^  [(0.19, 3.61), 0.03] | 0.15^a^  [(–0.76, 1.05), 0.75] | 0.32^a^  [(–0.55, 1.19), 0.47] |
| **Gender** | | | | | |
| Female | 2.34^b^  [(–5.98, 10.66), 0.58] | 9.33^b^  [(5.92, 12.74), <0.001] | –0.87^b^  [(–19.61, 17.88), 0.93] | 9.52^b^  [(–0.19, 19.24), 0.06] | 0.19^b^  [(–9.23, 9.61), 0.97] |
| Male | 0 | 0 | 0 | 0 | 0 |
| **Hypertension** | | | | | |
| Yes | 2.73^b^  [(–3.83, 9.29), 0.41] | 2.37^b^  [(–0.50, 5.24), 0.11] | 11.56^b^  [(–3.14, 26.27), 0.12] | 7.09^b^  [(–0.58, 14.76), 0.07] | 4.72^b^  [(–2.68, 12.12), 0.21] |
| No | 0 | 0 | 0 | 0 | 0 |
| **Type 2 diabetes** | | | | | |
| Yes | –0.26^b^  [(–8.43, 7.91), 0.95] | –4.37^b^  [(–7.91, –0.84), 0.02] | 21.07^b^  [(2.93, 39.21), 0.02] | –1.67^b^  [(–11.29, 7.94), 0.73] | 2.70^b^  [(–6.53, 11.93), 0.57] |
| No | 0 | 0 | 0 | 0 | 0 |
| **Smoking status** | | | | | |
| Current smoker | 0.95^b^  [(–6.81, 8.72), 0.81] | –2.03^b^  [(–5.36, 1.30), 0.23] | 10.55^b^  [(–6.75, 27.86), 0.23] | 1.99^b^  [(–7.13, 11.12), 0.67] | 4.02^b^  [(–4.75, 12.79), 0.37] |
| Ex-smoker | –1.35^b^  [(–9.51, 6.82), 0.75] | –5.58^b^  [(–9.08, –2.08), 0.002] | 18.23^b^  [(0.03, 36.42), 0.05] | –2.65^b^  [(–12.25, 6.95), 0.59] | 2.93^b^  [(–6.29, 12.15), 0.53] |
| Nonsmoker | 0 | 0 | 0 | 0 | 0 |
| **ACS type** | | | | | |
| STEMI | –2.07^b^  [(–10.55, 6.42), 0.63] | 1.31^b^  [(–2.42, 5.03), 0.49] | –9.99^b^  [(–29.16, 9.17), 0.31] | –1.08^b^  [(–11.07, 8.91), 0.83] | –2.38^b^  [(–12.01, 7.25), 0.63] |
| Non–STEMI | 4.11^b^  [(–4.49, 12.71), 0.35] | 2.81^b^  [(–0.96, 6.58), 0.14] | –8.00^b^  [(–27.43, 11.43), 0.42] | 5.69^b^  [(–4.44, 15.81), 0.27] | 2.88^b^  [(–6.88, 12.64), 0.56] |
| Unstable angina | 0 | 0 | 0 | 0 | 0 |
| **LLT received during study** | | | | | |
| Statin + Additional medication (ezetimibe or PCSK9i) | –5.28  [(–12.04, 1.49), 0.13] | –0.50  [(–3.49, 2.49), 0.74] | 3.75  [(–11.55, 19.06), 0.63] | –6.61  [(–14.57, 1.34), 0.11] | –6.11  [(–13.76, 1.54), 0.12] |
| Statin only | 0 | 0 | 0 | 0 | 0 |
| **Lipid profile components measured** **at ≤5 days from hospital admission** | | | | | |
| **LDL-C** | 0.10^a^  [(0.02, 0.18), 0.01] | 0.02^a^  [(–0.01, 0.05), 0.24] | –0.16^a^  [(–0.34, 0.01), 0.07] | 0.07^a^  [(–0.02, 0.16), 0.14] | 0.05^a^  [(–0.04, 0.14), 0.28] |
| **HDL-C** | 0.04^a^  [(–0.25, 0.33), 0.80] | 0.54^a^  [(0.44, 0.64), <0.001] | –1.00^a^  [(–1.63, –0.36), 0.002] | 0.40^a^  [(0.06, 0.74), 0.02] | –0.14^a^  [(–0.47, 0.18), 0.38] |
| **Triglycerides** | 0.00^a^  [(–0.05, 0.04), 0.98] | –0.04^a^  [(–0.06, –0.02), <0.001] | 0.34^a^  [(0.26, 0.42), <0.001] | 0.02^a^  [(–0.03, 0.07), 0.36] | 0.06^a^  [(0.01, 0.11), 0.01] |
| **Total cholesterol** | 0.08^a^  [(0.01, 0.014), 0.03] | 0.03^a^  [(0.00, 0.06), 0.03] | –0.04^a^  [(–0.19, 0.12), 0.62] | 0.09^a^  [(0.01, 0.17), 0.03] | 0.05^a^  [(–0.02, 0.13), 0.17] |
| **Non–HDL-C** | 0.08^a^  [(0.01, 0.15), 0.03] | 0.00^a^  [(–0.03, 0.03), 0.89] | 0.02^a^  [(–0.14, 0.18), 0.82] | 0.07^a^  [(–0.01, 0.15), 0.1] | 0.07^a^  [(–0.01, 0.15), 0.1] |
| **Lipid profile components measured** **at baseline** | | | | | |
| **LDL-C** | 0.42^a^  [(0.31, 0.54), <0.001] | –0.03^a^  [(–0.09, 0.03), 0.34] | 0.24^a^  [(–0.07, 0.55), 0.12] | 0.38^a^  [(0.23, 0.53), <0.001] | 0.41^a^  [(0.27, 0.56), <0.001] |
| **HDL-C** | –0.17^a^  [(–0.49, 0.15), 0.30] | 0.80^a^  [(0.72, 0.87), <0.001] | –1.18^a^  [(–1.90, –0.47), 0.001] | 0.33^a^  [(–0.05, 0.71), 0.08] | –0.46^a^  [(–0.83, –0.10), 0.01] |
| **Triglycerides** | 0.07^a^  [(0.00, 0.14), 0.04] | –0.07^a^  [(–0.10, –0.04), <0.001] | 0.83^a^  [(0.74, 0.92), <0.001] | 0.17^a^  [(0.09, 0.25), <0.001] | 0.24^a^  [(0.17, 0.31), <0.001] |
| **Total cholesterol** | 0.32^a^  [(0.20, 0.43), <0.001] | 0.04^a^  [(–0.01, 0.09), 0.14] | 0.60^a^  [(0.33, 0.87), <0.001] | 0.52^a^  [(0.40, 0.64), <0.001] | 0.48^a^  [(0.36, 0.60), <0.001] |
| **Non–HDL-C** | 0.34^a^  [(0.23, 0.44), <0.001] | –0.07^a^  [(–0.13, –0.02), 0.006] | 0.76^a^  [(0.51, 1.01), <0.001] | 0.47^a^  [(0.34, 0.59), <0.001] | 0.54^a^  [(0.43, 0.65), <0.001] |

ACS, acute coronary syndrome; BMI, body mass index; CI, confidence interval; HDL-C, high-density lipoprotein cholesterol; LDL-C, low-density lipoprotein cholesterol; LLT, lipid-lowering therapy; PCSK9i, proprotein convertase subtilisin/kexin type 9 inhibitor; STEMI, ST-elevation myocardial infarction.

^a^Indicates average change in the lipid component for a unit increase in the explanatory variable.

^b^Indicates average change in the lipid component compared with the reference category (i.e., category = 0).
